# Protein losing enteropathy after the Fontan operation

**DOI:** 10.1016/j.ijcchd.2022.100338

**Published:** 2022-01-26

**Authors:** Tarek Alsaied, Adam M. Lubert, David J. Goldberg, Kurt Schumacher, Rahul Rathod, David A. Katz, Alexander R. Opotowsky, Meredith Jenkins, Christopher Smith, Jack Rychik, Shahnawaz Amdani, Lizabeth Lanford, Frank Cetta, Christian Kreutzer, Brian Feingold, Bryan H. Goldstein

**Affiliations:** a Heart Institute, UPMC Children's Hospital of Pittsburgh, Division of Pediatric Cardiology, University of Pittsburgh School of Medicine, Pittsburgh, PA, USA; b Heart Institute, Department of Pediatrics, Cincinnati Children's Hospital Medical Center, University of Cincinnati, Cincinnati, OH, USA; c The Children's Hospital of Philadelphia, Division of Pediatric Cardiology, Perelman School of Medicine, Philadelphia, PA, USA; d Congenital Heart Center, C.S. Mott Children's Hospital, University of Michigan, Ann Arbor, MI, USA; e Department of Cardiology, Boston Children's Hospital and Department of Pediatrics, Harvard Medical School, Boston, MA, USA; f Division of Pharmacy, Cincinnati Children's Hospital Medical Center, Cincinnati, OH, USA; g Department of Pediatric Cardiology, Cleveland Clinic Children's Hospital, Cleveland, OH, USA; h Division of Pediatric Cardiology, Department of Cardiovascular Diseases, Mayo Clinic, Rochester, MN, USA; i Division of Pediatric Cardiovascular Surgery, Hospital Universitario Austral, Pilar, Buenos Aires, Argentina

**Keywords:** Fontan, Single ventricle, Protein losing enteropathy, Heart failure

## Abstract

The Fontan or Fontan Kreutzer procedure is the culmination of staged, surgical palliation of functional single ventricle congenital heart disease, offering the potential for survival and good quality of life well into adulthood. As more patients with Fontan circulation age, a variety of complications involving almost every organ system may occur. Protein-losing enteropathy is a major cause of morbidity and mortality after the Fontan operation, occurring more often in patients with adverse hemodynamics and presenting weeks to years after Fontan surgery. The causes are not well understood, but likely include a combination of lymphatic insufficiency, high central venous pressure, loss of heparan sulfate from intestinal epithelial cells, abnormal mesenteric circulation, and intestinal inflammation. A comprehensive evaluation including multimodality imaging and cardiac catheterization is necessary to diagnose and treat any reversible causes. In advanced cases, early referral for heart transplantation evaluation or lymphatic decompression procedures (if the single ventricle function remains adequate) is indicated. Despite the improvement in detection and management options, the mortality remains high. Standardization of protein-losing enteropathy definition and management strategies will help facilitate interpretation of research and clinical experience, potentially fostering the identification of new therapies. Based on the published data, this review suggests a standardized approach to diagnosis and treatment.

## Non-standard abbreviations and acronyms

Protein-losing enteropathy: PLE
Fecal α1 antitrypsin: A1AT

## 1 Introduction

The Fontan or Fontan Kreutzer procedure offers patients with functional single ventricle congenital heart lesions much improved survival and quality of life well into adulthood [1,2]. Indications for the Fontan operation have expanded over time to include more patients with functional single ventricle physiology and with incremental modifications resulting in better outcomes [3]. However, a variety of sequelae secondary to living with this physiology are becoming apparent [4]. Protein-losing enteropathy (PLE) is one of the most severe pathological manifestations of the Fontan circulation, and can be associated with Fontan circulatory failure [[5], [6], [7]]. In this manuscript, we review the pathophysiology, definition, prevalence, diagnosis, and management options for PLE. We suggest a management paradigm based on the available literature.

### 1.1 Pathophysiology, etiology and risk factors

**Fig. 1:**
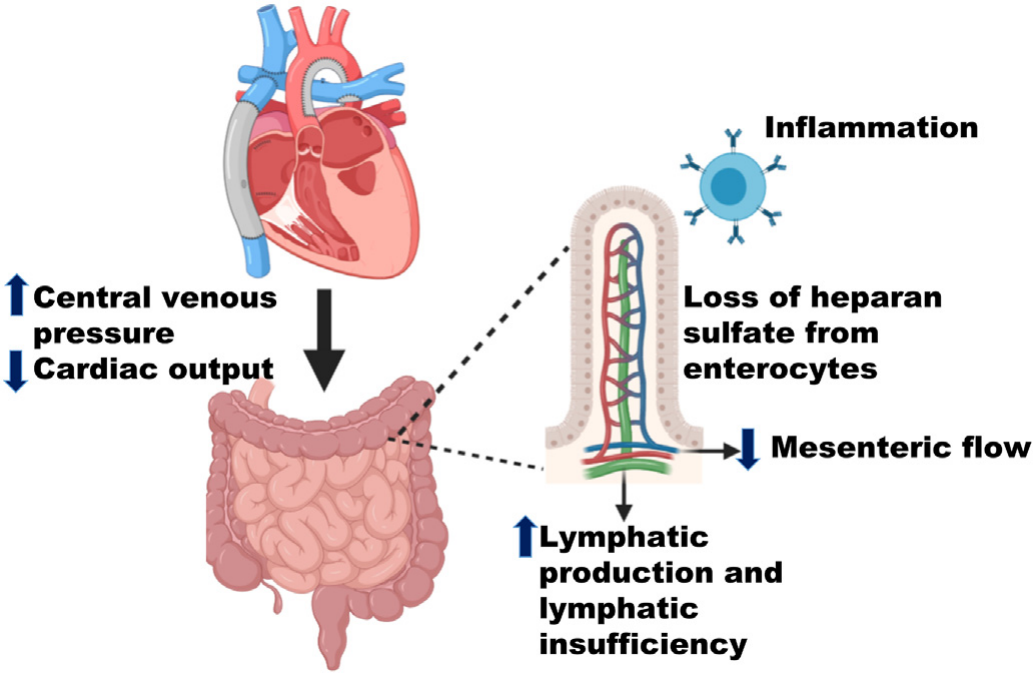
The pathophysiology of protein losing enteropathy in Fontan circulation.

Fontan-associated PLE results from excessive leakage of protein from the gastrointestinal system. The causes are not well understood, but likely include a combination of lymphatic insufficiency, high central venous pressure, loss of heparan sulfate from intestinal epithelial cells, abnormal mesenteric circulation, and intestinal inflammation (Fig. 1) [[7], [8], [9]]. Lymphatic insufficiency post-Fontan results from a combination of elevated pressure in the lymphatic system, increased lymph production, lymphatic vessel injury (trauma) from repeated thoracic surgery, and congenital or acquired abnormalities of the lymphatic circulation [10]. Because the lymphatic circulation drains to the systemic venous circulation via the thoracic duct, typically entering at the left brachiocephalic vein, elevated lymphatic pressure is universal post-Fontan as the central venous pressure is always elevated in this population. Elevated capillary pressure results in increased lymph production [11]. Chronic severe lymphatic overload may cause pathologic dilation and consequent dysfunction of the lymphangion, the functional unit of the lymphatic system. This leads to lymphatic proliferation due to increased lymphangiogenesis signaling and formation of abnormal intestinal lymphatic connections [12,13]. The loss of protein appears to be secondary to lymphatic distension and rupture of the lymphatic vessels of the small intestines resulting in lymphatic spillage into the intestinal lumen [9]. This pathophysiologic construct is supported by recent reports of dramatic improvement in PLE after diversion of the innominate vein (which drains the thoracic duct) to the lower-pressure systemic atrium, thereby decompressing the thoracic duct at the expense of right to left shunt and possible resultant systemic desaturation [14,15]. PLE can manifest in Fontan patients with acceptable hemodynamic profiles, suggesting pathologic (for Fontan circulation) central venous hypertension is not necessary for PLE to develop. This suggests that while an elevated central venous pressure is present in Fontan related PLE, central venous hypertension is not the sole cause, as the magnitude of pressure elevation is not associated with PLE development or extent of PLE related symptoms [[16], [17], [18]]. An important consideration is that with reduced serum protein levels in PLE, the oncotic pressure is also substantially reduced resulting in edema and less intravascular volume [17]. Thereby, venous pressures, pulmonary artery pressures and systemic ventricular pressures assessed during catheterization may be “artificially” low due to decreased protein levels in Fontan PLE patients [17].

Other factors may also be implicated in the development of PLE. A subset of PLE patients have diminished heparan sulfate, a polysaccharide involved in protein trafficking from the enterocyte membrane, resulting in protein loss [19,20]. Additionally, low cardiac output and elevated mesenteric vascular resistance potentially result in chronic bowel ischemia, which has been described in PLE [16,21,22]. Another proposed mechanism is small intestinal inflammation with increased mucosal interferon γ and mononuclear infiltrates [21,23]. All of the above mechanisms lead to leakage and loss of lymphatic fluids into the intestinal lumen. Why some patients develop PLE and others do not remains unclear. To date, no genetic factors or changes in the intestinal microbiome that predispose to PLE have been described. It is possible that there are natural variations in the lymphatic system architecture such that only a subset of individuals have unique anatomical connections with potential for abundant drainage and decompression into the gut lumen. In addition, the lymphatic system in Fontan patients is working near maximum capacity, such that a relatively minor trigger, including infection, can generate enteric inflammation which may lead to lymphatic rupture in some predisposed patients [15,[24], [25], [26], [27]]. Once PLE starts, it can be self-sustaining; this is in part secondary to the enhanced lymph production from decreased oncotic pressure due to hypoalbuminemia [15]. Additionally, PLE results in intestinal wall edema and further protein loss.

Reported risk factors for PLE include: older age at Fontan, hypoplastic left heart syndrome, atriopulmonary type Fontan, post-operative chylothorax and/or prolonged pleural drainage (at stage II palliation or Fontan completion surgery), elevated Glenn pressure, elevated atrial pressure post Fontan, and arrhythmia [[28], [29], [30]].

### 1.2 PLE definition

Various definitions of PLE have been used, perhaps due to its variable course and nature [31]. Having a standardized definition and a set of diagnostic criteria for PLE is critical to understanding its prevalence and the effect of different treatment strategies. A recent consensus statement, using the modified Delphi methodology, on Fontan related morbidities adopted the following definition of PLE [31,32]:

“PLE is the state of increased enteric protein loss [as measured by fecal α1 antitrypsin (A1AT) (spot >54 mg/dl, α1 antitrypsin clearance >27 ml /24 hours without diarrhea and >56 ml/24 hours with diarrhea)]. It can be subclinical or associated with 1) hypoalbuminemia <3.5 g /dl and total protein <6 g/dl, and 2) any of the following clinical symptoms: edema, abdominal distention or discomfort, diarrhea, or effusions (ascites, pleural or pericardial effusions).”

### 1.3 PLE prevalence

The prevalence of Fontan-associated PLE is thought to be around 5% and can occur at any point after the Fontan operation [10,33]. A recent study evaluated the prevalence of PLE in 1561 patients from the Australia and New Zealand (ANZ) Fontan Registry and found 45 patients (∼3%) with PLE. Median time from Fontan to onset of PLE was 5 years. The 5-year freedom from death or transplantation was 70% [29].

This prevalence is less than previous studies, but the 5-year survival after diagnosis of PLE is improved compared to earlier studies. In a 1998 multicenter study, 5-year survival after diagnosis of PLE was only 50% [5]. In a more recent review of the Mayo Clinic Fontan database, published in 2015, 5-year survival remained only 50% (21). However, in a non-cohort sample of that database from patients enrolled after 1992, 5-year survival after diagnosis of PLE had improved to 88% (5). The implication was that more recent treatment options and heightened surveillance may have improved outcomes. Differences in prevalence and survival data may be influenced by referral bias at single center versus multicenter population-based cohorts. They may also be influence by different definitions of PLE in earlier studies. Going forward, using a consistent definition will facilitate direct comparison between studies and registries (27).

### 1.4 PLE presentation

Many patients may report viral-like symptoms (e.g. fever and malaise) 1–3 weeks before the onset of PLE [34]. Symptoms of PLE may include: peripheral edema, abdominal distention due to ascites, pleural effusion, and pericardial effusion. Subtle periorbital and facial edema are often the initial presentation, frequently occurring in the morning after waking, as these tissues are highly sensitive to reduced oncotic pressure. Diarrhea may be present, but chronic diarrhea has been reported in only 11–20% of patients [5,30]. Chronic protein and other nutrient losses increase the risk of malnutrition which can present as poor wound healing and easy bruising [33]. In younger children this may also manifest as growth failure. Additionally, vitamin D deficiency and hypocalcemia can be seen and may be associated with osteoporosis [33]. In severe cases, coagulation abnormalities due to enteric loss of both pro and anti-clotting factors can manifest as thrombosis or bleeding [33].

### 1.5 Diagnosis

Providers who are caring for Fontan patients should have a high index of suspicion for PLE if any of the above symptoms develop. Screening for PLE by laboratory surveillance of serum albumin and/or total protein levels and fecal A1AT is suggested every 3–4 years in children below the age of 12 years and every 1–3 years in adolescents and adults [35]. A subset of patients will have subclinical PLE with normal serum albumin and total protein levels but elevated fecal A1AT. Whether subclinical PLE is a stage that precedes PLE is some or all cases is yet to be determined. Thus, patients with subclinical PLE may benefit from more frequent screening for PLE [15].

The diagnosis of PLE is made when patients have hypoalbuminemia/hypoproteinemia and evidence of elevated fecal A1AT [34]. Treatment commences with the diagnosis of PLE, as detailed in the treatment protocol suggested below. Supplemental Fig. 1 summarizes the approach used for diagnosing PLE.

## 2 Treatment options

Here we summarize the different treatment options in patients with PLE. We will then present a proposed stepwise approach to treating patients with PLE [7]. The first step in managing patients with PLE includes a careful evaluation of any modifiable anatomic or hemodynamic derangements. The evaluation should include an: echocardiogram, cross-sectional imaging (CT or MRI including T2 lymphangiography), electrocardiogram, exercise testing, Holter monitor, and cardiac catheterization with careful hemodynamic assessment and comprehensive angiography. Optimizing treatment for ventricular dysfunction and arrhythmia (including sinus node dysfunction) may improve PLE symptoms [33,34]. Screening for sleep apnea may be necessary particularly in adults based on limited data [6,36,37]. Additionally, addressing any anatomic obstruction in the Fontan pathway, typically achieved through transcatheter techniques, should be undertaken to minimize impedance with a goal to decrease central venous pressure and venous congestion, while maximizing cardiac output [34]. In patients with elevated pulmonary vascular resistance, or those with central venous hypertension and/or low cardiac output, a pulmonary vasodilator can be considered (as detailed below).

### 2.1 Supportive care and nutrition

Nutritional support is an important part of treating PLE [33,38]. Iron deficiency is common in Fontan patients and thus PLE may improve with iron replacement [38]. The mechanism by which iron replacement may improve PLE is not well known, but iron therapy may be associated with decreased proinflammatory cytokines [39,40]. Additionally, correcting anemia may improve diastolic dysfunction and thus lower Fontan pressure [41]. PLE is associated with an increased risk for bone demineralization related to the disease process itself and to side effects from treatment with steroids or heparin. Replacement of fat-soluble vitamins (vitamin A,D, E and K), particularly vitamin D, may be required due to impaired absorption of these fat-soluble vitamins [42]. A high protein (>2 ​g/kg/day) and low fat (<25% of caloric intake) diet, while increasing medium-chain triglycerides (MCT) intake, is recommended as absorption of MCT does not depend on the intestinal lymphatic system and thus reduces lymphatic production [33,34].

### 2.2 Medications

#### 2.2.1 Diuretics and albumin infusions

Diuretics provide symptomatic relief for edema associated with PLE-related hypoalbuminemia and are a cornerstone of symptomatic management. Diuresis, frequently with loop diuretics, is tailored to patient response. Intravenous diuresis may be needed in some patients, particularly with very low serum albumin levels, ascites, and intestinal edema where oral absorption of medications may be impaired. Direct replacement of serum albumin by infusion is not a feasible long-term treatment strategy, as it only provides short-term symptomatic relief and does not reverse the underlying pathophysiology. Further, albumin infusion typically requires hospitalization or outpatient infusion within a hospital setting, along with intravenous access, thereby limiting its frequency of administration [33]. However, in some patients with severe volume overload and hypoalbuminemia, the addition of albumin to loop diuretics significantly enhances diuresis. In these cases, our experience is that the albumin supplementation needs to be frequent with studies suggesting 1 ​g/kg every 4 ​h with intravenous diuretics to achieve effective diuresis [43] (Table 1).

**Table 1:** Dosing and monitoring of common medications used in the treatment of PLE.

Medication	Dose	Monitoring
Furosemide or other loop diuretics	1 ​mg/kg IV or PO every 6–24 ​h	Electrolytes, fluid status
	When kidney function is normal, a 40 ​mg dose of furosemide is approximatey equal to 1 ​mg of bumetanide and 20 ​mg of torsemide.	
Albumin (25%)	1 ​g/kg IV as needed, as often as every 4–6 ​h	Fluid status
Budesonide	Starting dose 6 ​mg–9 ​mg PO per day, typically divided three times daily..	Adrenal suppression, Cushingoid features, hypertension
Spironolactone^a^	1–4 ​mg/kg per day (Max 200 ​mg per day)	Hyperkalemia, gynecomastia
Sildenafil^b^	1 ​mg/kg (Max 20 ​mg per dose) PO three times daily	Flushing, priapism, hypotension
Midodrine	1.25 ​mg–10 ​mg PO two to three times daily	Hypertension
Dopamine	Short term infusion at 3–5 mcg/kg/min	Arrhythmia and hemodynamics
Heparin (unfractionated)	3000–5000 unit/m^2^/day subcutaneous	Bleeding and osteopenia
Octreotide	1-4 mcg/kg subcutaneous every 1–2 days	Gastrointestinal side effect and musculoskeletal pain

a Consider eplerenone if side effects from spironolactone.

b Other phosphodiesterase inhibitors and other pulmonary vasodilator classes can be used. PO: by mouth.

Spironolactone is commonly used as a weak diuretic in addition to its potassium-sparing benefits in patients also receiving loop diuretics. Interestingly, remission of PLE has been reported in patients treated with relatively high-dose spironolactone [[44], [45], [46]]. The mechanism of action is thought to be related to possibly decreasing intestinal protein loss and improving lymphatic transit. Additionally, aldosterone antagonism has an overall positive effect on the cardiovascular system and decreases the aldosterone-mediated fluid retention [33,47,48].

#### 2.2.2 Corticosteroid therapy

Corticosteroid therapy may help decrease the permeability of the intestinal epithelium resulting in decreased enteric protein loss and normalization of serum albumin and total protein concentrations [49]. Corticosteroids also suppress inflammation which is a driver of PLE. Historically, the use of prednisone resulted in many adverse effects due to systemic absorption [33]. Budesonide has enteric-specific effects with 90% deactivation rate by the intestine and liver; resulting generally in fewer systemic adverse effects [33]. In a recent meta-analysis, the use of budesonide in patients with PLE following Fontan was found to be associated with a significant increase in serum albumin levels [50]. Budesonide in combination with pulmonary vasodilator therapy may also be associated with improved survival [51]. Despite the lower systemic absorption of budesonide, signs and symptoms of supraphysiologic corticosteroid exposure are common including the development of Cushingoid features, suppressed cortisol levels, and growth failure [52]. Side effects are more common in older patients [6,53]. This may be secondary to impaired hepatic metabolism from Fontan-associated liver disease [52]. Careful evaluation of liver function is warranted in PLE.

#### 2.2.3 Phosphodiesterase-5 inhibitors (PDE5i)

Although efficacy is not well established, sildenafil has been used successfully in patients with PLE [49,54]. Use of other PDE5i have been less commonly reported, though there is no reason to think the effects would differ; and it is plausible that other pulmonary vasodilator classes would have similar impact [55,56]. The benefit in patients with PLE may be a result of decreased Fontan pressure and mesenteric venous congestion through a reduction in pulmonary vascular resistance. Sildenafil may also reduce mesenteric venous congestion by direct reduction of mesenteric vascular resistance [57]. Thus, pulmonary vasodilators are often prescribed in PLE, especially when there is elevated pulmonary vascular resistance [35]. It is important to note that the central venous pressure and pulmonary vascular resistance measured by cardiac catheterization may be artificially low in PLE as noted above [17].

#### 2.2.4 Midodrine

One case series described the use of midodrine being associated temporally with improvement in PLE that had been refractory to standard medical treatments [58]. The proposed mechanism is through α-adrenergic stimulation, which may increase lymphatic tone [58].

#### 2.2.5 Heparin

Several case reports have shown that subcutaneous heparin may lead to symptomatic and laboratory improvement in a small subset of patients with PLE [[59], [60], [61], [62]]. In most patients, however, heparin therapy is ineffective [51,61]. As with other therapies, the mechanism is not fully understood. Heparin may decrease the permeability of the endothelial membrane and reduce protein leakage. Importantly, chronic use of heparin is associated with the development of osteopenia. Bone mineral density may be less impacted by low molecular weight heparin (LMWH) [51,59,61]. However, there is little evidence for LWMH efficacy in PLE, and the study results are contradictory [62]. A trial of heparin is reasonable for PLE patients not responsive to alternative therapies, but if there is no improvement after a few weeks, discontinuation is recommended given the risk of adverse effects.

#### 2.2.6 Dopamine

Dopamine infusion has been used for treatment of refractory severe PLE as well as a bridge to transplantation with resultant marked improvement in protein loss in a few reported cases [63]. Hypothetical mechanisms include increased contractility of the lymphatic vessels and increased mesenteric blood flow [63].

#### 2.2.7 Octreotide

Octreotide has also been used in severe cases and mimics the effects of somatostatin. Octreotide may decrease the absorption of triglycerides resulting in decreased lymphatic formation and thus less overall lymphatic leak through the intestines [64]. Common side effects include gastrointestinal intolerance, cholelithiasis, and musculoskeletal pain [64]. While there is a theoretical benefit to the use of octreotide, anecdotally it does not provide a significant sustained clinical improvement.

### 2.3 Anatomic interventions

Catheter-based interventions anticipated to lower venous pressure should be considered. Any obstruction in the Fontan circuit, including the Fontan conduit or baffle/tunnel, central veins, pulmonary arteries and veins should be addressed [34,65]. Of note, many patients with Fontan circulation may have no gradient across the relatively low-pressure Fontan venous circuit despite anatomical stenosis. Given the challenges in effectively treating PLE, addressing “minor” anatomical stenosis is commonly felt to be warranted. If the Fontan pressure is elevated, and not caused by any anatomical obstruction, a fenestration can be created within the Fontan circuit to provide a pop-off to the lower-pressure pulmonary venous atrium creating a right to left shunt and thereby augmenting systemic ventricular preload and, thus, cardiac output [65,66]. Fenestration is accompanied by cyanosis, increased risk of stroke, as well as spontaneous fenestration closure (often with acute PLE recurrence). Even with the use of systemic anticoagulation and fenestration stent, long-term maintenance of transcatheter Fontan fenestration patency is frequently challenging (51). Thus the effectiveness of a fenestration creation in PLE is limited.

Fontan takedown to a Glenn circulation or aortopulmonary shunt can also be used in selected cases. This option is used only in cases when medical and interventional therapies have failed to resolve PLE, heart transplantation is not possible, or when PLE occurs in the context of acute Fontan failure (e.g., large volume chylous effusions) and an urgent intervention is warranted [67]. These procedures are associated with high mortality especially when performed more than 6 months after the Fontan operation and should be used only as a last resort [68]. Also, Lymphatic decompression via innominate vein turn-down procedure may play an important role in this setting [69].

Assessment for diaphragmatic palsy and diaphragmatic plication can result in significant improvement in some patients with PLE as impaired ventilation represents a severe obstacle to the passive lung perfusion in Fontan patients (25, 41).

Other novel surgical approaches include a surgical procedure to restore a physiological gradient between the portal vein and the inferior vena cava by connecting the hepatic veins to the atrium [70].

### 2.4 Heart transplantation

Heart transplantation is generally thought to provide a “cure” for PLE in selected Fontan patients. A recent multi-institutional study from 12 centers in the United States showed that PLE resolves in nearly all transplant survivors although it may take up to one year post-transplant for the PLE to completely resolve [71]. While some studies show that PLE severity, duration, and treatment did not influence post-transplant outcomes, other studies have reported up to two-fold increase in mortality post-transplant in patients with PLE [[71], [72], [73], [74]].

Although transplantation frequently offers benefit for individuals with Fontan circulation and PLE, the presence of antibodies directed against donor antigens (allosensitization) is common [71] and often complicates waitlist and transplant outcomes [75]. Anecdotally, some patients with PLE who test negative for donor-specific antibodies prior to transplantation abruptly turn positive in the days and weeks following transplantation, often in the context of antibody-mediated rejection. This could be related to the chronic hypogammaglobulinemia seen in patients with PLE (due to enteric loss) before transplant that may serve to mask allosensitization [76].

### 2.5 Lymphatic imaging and interventions

Imaging the lymphatic system remains challenging. T2-weighted magnetic resonance imaging (MRI) techniques can be used to provide good visualization of the thoracic duct [77]. Additionally, grading of lymphatic abnormalities before the Fontan operation based on quantification of the extent of lymphatic distention by MRI was proposed to predict patients with worse post-Fontan outcomes [78]. Higher grade lymphatic circulation abnormalities are associated with early Fontan failure [78]. In PLE, this technique shows larger sizes of the thoracic duct compared to Fontan patients without PLE [79]. Despite the good spatial resolution of nonenhanced T2 weighted MRI, it does not provide physiologic information about lymphatic flow and often fails to identify the anatomic location of lymphatic leaks [80]. Dynamic contrast-enhanced MR lymphangiography (DCMRL) uses gadolinium-based contrast injected in the lymph nodes and tracks the lymphatic flow over time to detect physiologic flow abnormalities and localize lymphatic leaks [77,80]. However, intranodal DCMRL does not allow visualization of hepatic or mesenteric lymphatic vessels and often does not show the origin of enteric protein loss.

Liver lymphangiography with fluoroscopy and more recently with intrahepatic DCMRL, showed dilated hepatic lymphatics and hepatoduodenal connection in patients with PLE [81,82]. In one study, injection of dye into liver lymphatics confirmed lymphatic leak in the duodenum in the majority of patients with PLE [8] and was recently highly associated with PLE in another larger cohort [82]. Nevertheless, intrahepatic connections are not the only source identified in patients with PLE. With advances in DCMRL techniques, visualization of mesenteric lymphatics have been developed and demonstrated mesenteric-duodenal connections that can also result in enteric protein loss further illustrating the complexity of anatomic causes in PLE [83].

With improved understanding of lymphatic anatomy and flow, new catheter-based lymphatic interventions to embolize abnormal channels are being utilized with selective embolization of abnormal hepatic and periduodenal lymphatics [8]. These interventions have shown promise in a small series of patients with temporary or sustained improvement in albumin in 6 out of 8 patients, although 2 patients had major gastrointestinal bleeding post intervention [8,84]. More published data, refinement of lymphatic interventional techniques and scaling to multiple treating centers will be needed to determine long-term outcomes and the role of this treatment pathway in PLE [8,85].

In addition to direct lymphatic interventions, other anatomic interventions targeting the increased lymphatic pressures/afterload have been described. Prominent among these is anatomic diversion of the innominate vein to the systemic atrium, which has been described using both transcatheter and surgical techniques [[86], [87], [88]]. These techniques have shown improvement in PLE symptoms in a small series of patients, but long-term outcomes and consequences of creating a right to left shunt have not yet been determined [69,87,89]. This newer interventional strategy for PLE has theoretical appeal, as it targets the venous decompression (right to left shunt creation) at the thoracic duct insertion site, as opposed to Fontan fenestration creation which also generates right to left shunt but is less focused on decompression of the lymphatic circulation. Interestingly, it has also been applied as a tailored strategy for patients with thoracic lymph abnormalities detected in routine pre-Fontan T2 MRI with promising results [88].

As a rule, all these new techniques require an acceptable function of the single ventricle. Patients with moderate to severe ventricular dysfunction will be better served with heart early referral transplantation.

## 3 A proposed approach to PLE management

We propose the following approach to management of patients with PLE after Fontan recognizing that an individualized approach to each patients is critical based on patient's anatomy and physiology [90]. These suggestions reflect a thorough review of the literature and the informal consensus of this writing group. It is important to recognize that the data behind these suggestions is based on observational studies with small sample size rather than controlled trials [91].

In asymptomatic patients with subclinical PLE (increased fecal A1AT and no hypoalbuminemia), perform a careful invasive and noninvasive hemodynamic evaluation. If that evaluation demonstrates no issues that require intervention, then close monitoring for the development of clinical PLE is reasonable.

**Fig. 2:**
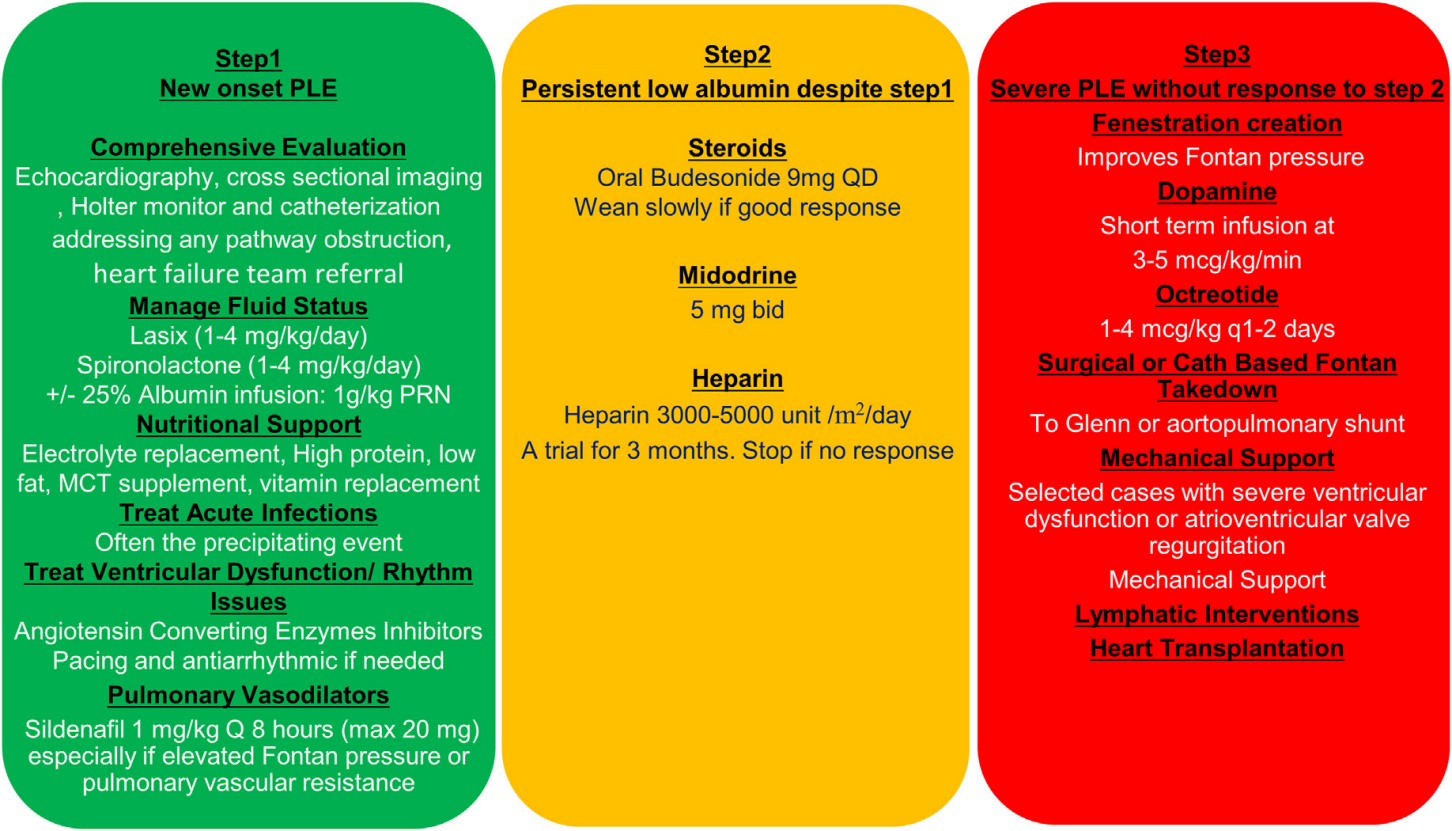
A suggested stepwise approach to managing a symptomatic patient with new diagnosis of protein losing enteropathy. When a diagnosis of protein losing enteropathy is confirmed, our approach is to start with step 1 therapies. If albumin concentration does not normalize with these interventions in 1–2 months, then transition to step 2. In more severe cases without response to step 2, transition to step 3. This approach is usually individualized to each patient based on response to therapy. ACEi: Angiotensin converting enzymes inhibitor, ARB: Angiotensin II receptor blocker, MCT: medium chain triglycerides.

When a diagnosis of PLE is confirmed, start with nutritional support using a high protein, high MCT and low-fat diet. Symptomatic treatment using diuretics (with or without albumin infusions) for signs of edema is warranted. A comprehensive anatomic and physiologic evaluation should be performed. Any identified anatomic pathology within the Fontan circuit (e.g. extracardiac conduit or branch pulmonary artery stenosis), or within the systemic circulation (e.g. atrial septal restriction, recurrent coarctation of the aorta) should be satisfactorily resolved through transcatheter or surgical interventions. Treatment with pulmonary vasodilator therapy should be considered especially in those with an elevated transpulmonary gradient or pulmonary vascular resistance, and in the absence of contraindications. Additionally, ventricular dysfunction or cardiac rhythm disturbances should be actively managed (Fig. 2).

If the albumin level does not normalize with these interventions in 1–3 months, budesonide should be started. If there is a sustained response to budesonide (∼6 months) with a stable albumin level and no symptoms, it can be weaned very slowly to the smallest dose to achieve clinical response. Caution is advised as there are reports of “loss of capture” (symptomatic PLE re-emergence) following budesonide wean with the subsequent inability to re-achieve disease quiescence on escalated dosing [49]. If there is no response to budesonide within a few months, it is important to slowly wean it off as prolonged exposure to corticosteroids may pose additional risk without benefit. Midodrine and heparin may also be trialed briefly as they have shown to improve symptoms in some patients and are relatively well tolerated. In the absence of benefit, they should not be continued.

In more severe cases where there is inadequate response to the above approach, the use of dopamine and octreotide may be trialed as a bridge to further therapies. A fenestration creation or lymphatic decompression via innominate vein turn down should be considered. Heart transplant evaluation and listing, if eligible, should not be delayed. Fontan takedown to a Glenn or aortopulmonary shunt, or newer targeted anatomic interventions such as innominate vein re-routing, can also be considered in some patients especially where the systemic ventricle has preserved function, or transplantation is not an option, or if an intervention is needed acutely to stabilize the clinical status. We recognize that as the understanding of the role of lymphatics progresses, the management strategy may yet evolve.

## 4 PLE course and prognosis

**Fig. 3:**
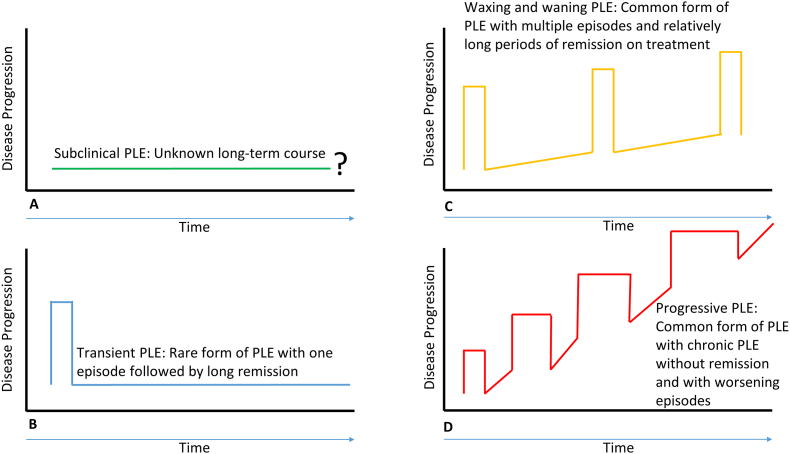
Conceptual framework for the course of PLE. A: Some patients have subclinical PLE which may or may not progress to overt PLE. B: Transient PLE with one episode followed by long term remission without long-term medical therapy has been reported. C: Waxing and waning course is common with multiple episodes followed by long periods of remission on medical therapy has been described. D: Progressive PLE with a chronic course of PLE, multiple worsening episodes and steep clinical decline is also reported.

PLE is often progressive, but the clinical course varies widely (Fig. 3). It remains difficult, if not impossible, to predict the future course for a given patient early in the disease course. In some, the severity waxes-and-wanes and complete remission can occur, while others may have only persistent mild hypoalbuminemia [90]. Transient PLE has also been described, with an episode of PLE followed by a long period of normal albumin and no symptoms.

The natural history of subclinical PLE (i.e., documented enteric protein loss with normal serum albumin and no symptoms) is not well defined and since it may precede clinical PLE, patients should be followed closely and monitored for the development of hypoalbuminemia. In addition, malnutrition may occur due to low-grade protein loss, even with normal serum albumin. Fig. 3 summarizes the clinical course of PLE. Ideally a more aggressive treatment approach should be implemented in patients with progressive PLE.

There are some patients where PLE can be controlled on corticosteroid therapy but relapses when the dose is weaned. The side effects of long-term treatment with corticosteroids should be closely monitored and careful evaluation for timing of transplantation is warranted. Early referral to heart failure/transplant team is warranted to start discussing the appropriate timing for listing in these patients [53].

## 5 Future directions

PLE represents an example where understanding the mechanism may require an integrated knowledge of the genetic factors, the patient specific phenotype and the patient environment [92]. Given the heterogeneity in patients with PLE, international collaborations and sharing biologic and phenotypic data among pediatric and adult institutions will be needed to make breakthrough discoveries [93]. The Australian and New Zealand Fontan Registry, started in 2009, has successfully generated foundational knowledge at the population level and a national registry in the United States is underway [94]. Additionally, the heterogeneity in PLE calls for an individualized approach to each patient. Once more data become available from the above collaboratives, it can be used to guide management and individualize care. Finally, educating and empowering the patients and families is important in congenital heart disease and particularly in patients with PLE as a chronic condition that requires long term care [95]. Interventions for education and empowering the families will need to be validated in PLE [92,95].

Finally, including lymphatic decompression as part of the Fontan completion procedure in selected patients may prevent PLE development, but its feasibility and efficacy will not become clear without the performance of a randomized clinical trial [88,96].

## 6 Summary

PLE is one of the more devastating and chronic morbidities after the Fontan operation. In this review, we summarize the etiology, risk factors, and diagnostic approach. We also propose a standardized approach to diagnosis and treatment of PLE based on the available literature. Standardization of the diagnostic and treatment approaches will allow direct comparison of different therapies and result in better management of patients with PLE.

## Funding

No authors report any funding related to the diagnosis or treatment of protein losing enteropathy. ARO has received research grant support (Actelion) and serves on an Independent Data Monitoring Committee for a clinical trial of pulmonary vasodilators in the Fontan circulation (Janssen Pharmaceuticals). BHG is a consultant for Mezzion Pharma.

## Disclosure

None.

## Declaration of competing interest

The authors declare that they have no known competing financial interests or personal relationships that could have appeared to influence the work reported in this paper.

## Appendix A Supplementary data

Multimedia component 1 Multimedia component 1

figs1 figs1

figs2 figs2

## References

[1] Fontan F., Baudet E. (1971). Surgical repair of tricuspid atresia. Thorax.

[2] Kreutzer G., Galíndez E., Bono H. (1973). An operation for the correction of tricuspid atresia. J Thorac Cardiovasc Surg.

[3] d'Udekem Y., Iyengar A.J., Cochrane A.D. (2007). The Fontan procedure: contemporary techniques have improved long-term outcomes. Circulation.

[4] Gordon-Walker T.T., Bove K., Veldtman G. (2019). Fontan-associated liver disease: a review. J Cardiol.

[5] Mertens L., Hagler D.J., Sauer U. (1998). Protein-losing enteropathy after the Fontan operation: an international multicenter study. PLE study group. J Thorac Cardiovasc Surg.

[6] John A.S., Johnson J.A., Khan M. (2014). Clinical outcomes and improved survival in patients with protein-losing enteropathy after the Fontan operation. J Am Coll Cardiol.

[7] Rychik J. (2007). Protein-losing enteropathy after Fontan operation. Congenit Heart Dis.

[8] Itkin M., Piccoli D.A., Nadolski G. (2017). Protein-losing enteropathy in patients with congenital heart disease. J Am Coll Cardiol.

[9] Rao P.S. (2007). Protein-losing enteropathy following the Fontan operation. J Invasive Cardiol.

[10] Meadows J., Jenkins K. (2011). Protein-losing enteropathy: integrating a new disease paradigm into recommendations for prevention and treatment. Cardiol Young.

[11] Sakai T., Yabuki S., Chang K. (1985). Effect of increased systemic venous pressure on thoracic duct and peripheral lymph flow in dogs. Lymphology.

[12] Menon S., Chennapragada M., Ugaki S. (2017). The lymphatic circulation in adaptations to the fontan circulation. Pediatr Cardiol.

[13] Veldtman G.R., Opotowsky A.R., Wittekind S.G. (2017). Cardiovascular adaptation to the Fontan circulation. Congenit Heart Dis.

[14] António M., Gordo A., Pereira C. (2016). Thoracic duct decompression for protein-losing enteropathy in failing fontan circulation. Ann Thorac Surg.

[15] Levitt D.G., Levitt M.D. (2017). Protein losing enteropathy: comprehensive review of the mechanistic association with clinical and subclinical disease states. Clin Exp Gastroenterol.

[16] Backer C.L. (2017). Rescuing the late failing fontan: focus on surgical treatment of dysrhythmias. Semin Thorac Cardiovasc Surg Pediatr Card Surg Annu.

[17] Simpson D.E. (2014). No central venous pressure protein losing enteropathy relation? Blame the albumin. Eur J Cardio Thorac Surg.

[18] Ohuchi H., Yasuda K., Miyazaki A. (2013). Haemodynamic characteristics before and after the onset of protein losing enteropathy in patients after the Fontan operation. Eur J Cardio Thorac Surg.

[19] Bode L., Freeze H.H. (2006). Applied glycoproteomics--approaches to study genetic-environmental collisions causing protein-losing enteropathy. Biochim Biophys Acta.

[20] Bode L., Murch S., Freeze H.H. (2006). Heparan sulfate plays a central role in a dynamic in vitro model of protein-losing enteropathy. J Biol Chem.

[21] Rychik J., Gui-Yang S. (2002). Relation of mesenteric vascular resistance after Fontan operation and protein-losing enteropathy. Am J Cardiol.

[22] Ostrow A.M., Freeze H., Rychik J. (2006). Protein-losing enteropathy after fontan operation: investigations into possible pathophysiologic mechanisms. Ann Thorac Surg.

[23] Shimizu T., Nagata S., Fujii T. (2003). Enhanced production of interferon-gamma as a possible cause of protein-losing enteropathy after modified Fontan operation. J Pediatr Gastroenterol Nutr.

[24] Kreutzer J., Kreutzer C. (2017). Lymphodynamics in congenital heart disease: the forgotten circulation. J Am Coll Cardiol.

[25] Connor F.L., Angelides S., Gibson M. (2003). Successful resection of localized intestinal lymphangiectasia post-Fontan: role of (99m)technetium-dextran scintigraphy. Pediatrics.

[26] Lenz D., Hambsch J., Schneider P. (2003). Protein-losing enteropathy in patients with Fontan circulation: is it triggered by infection?. Crit Care.

[27] Kreutzer C., Kreutzer G. (2017). The lymphatic system: the achilles heel of the fontan-Kreutzer circulation. World J Pediatr Congenit Heart Surg.

[28] Pundi K.N., Johnson J.N., Dearani J.A. (2015). 40-Year follow-up after the fontan operation: long-term outcomes of 1,052 patients. J Am Coll Cardiol.

[29] Sharma V.J., Iyengar A.J., Zannino D. (2021). Protein-losing enteropathy and plastic bronchitis after the Fontan procedure. J Thorac Cardiovasc Surg.

[30] Schumacher K.R., Stringer K.A., Donohue J.E. (2015). Fontan-associated protein-losing enteropathy and plastic bronchitis. J Pediatr.

[31] Udink Ten Cate F.E., Hannes T., Germund I. (2016). Towards a proposal for a universal diagnostic definition of protein-losing enteropathy in Fontan patients: a systematic review. Heart.

[32] Alsaied T., Rathod R.H., Aboulhosn J.A. (2021).

[33] Johnson J.N., Driscoll D.J., O'Leary P.W. (2012). Protein-losing enteropathy and the Fontan operation. Nutr Clin Pract.

[34] Rychik J., Spray T.L. (2002). Strategies to treat protein-losing enteropathy. Semin Thorac Cardiovasc Surg Pediatr Card Surg Annu.

[35] Rychik J., Atz A.M., Celermajer D.S. (2019 Jul 1). Evaluation and management of the child and adult with fontan circulation: a scientific statement from the American heart association. Circulation.

[36] Veldtman G.R., Webb G.D. (2014). Improved survival in Fontan-associated protein-losing enteropathy. J Am Coll Cardiol.

[37] Watson N.F., Bushnell T., Jones T.K. (2009). A novel method for the evaluation and treatment of obstructive sleep apnea in four adults with complex congenital heart disease and Fontan repairs. Sleep Breath.

[38] Yetman A.T., Everitt M.D. (2011). The role of iron deficiency in protein-losing enteropathy following the Fontan procedure. Congenit Heart Dis.

[39] Weiss G., Meusburger E., Radacher G. (2003). Effect of iron treatment on circulating cytokine levels in ESRD patients receiving recombinant human erythropoietin. Kidney Int.

[40] Tomkiewicz-Pajak L., Plazak W., Kolcz J. (2014). Iron deficiency and hematological changes in adult patients after Fontan operation. J Cardiol.

[41] Silverberg D.S., Wexler D., Iaina A. (2006). The interaction between heart failure and other heart diseases, renal failure, and anemia. Semin Nephrol.

[42] Goldberg D.J., Dodds K., Avitabile C.M. (2012). Children with protein-losing enteropathy after the Fontan operation are at risk for abnormal bone mineral density. Pediatr Cardiol.

[43] Dharmaraj R., Hari P., Bagga A. (2009). Randomized cross-over trial comparing albumin and frusemide infusions in nephrotic syndrome. Pediatr Nephrol.

[44] Okano S., Sugimoto M., Takase M. (2016). Effectiveness of high-dose spironolactone therapy in a patient with recurrent protein-losing enteropathy after the fontan procedure. Intern Med.

[45] Ringel R.E., Peddy S.B. (2003). Effect of high-dose spironolactone on protein-losing enteropathy in patients with Fontan palliation of complex congenital heart disease. Am J Cardiol.

[46] Grattan M.J., McCrindle B.W. (2010). Recurrent exacerbations of protein-losing enteropathy after initiation of growth hormone therapy in a Fontan patient controlled with spironolactone. Congenit Heart Dis.

[47] Telinius N., Baandrup U., Rumessen J. (2014). The human thoracic duct is functionally innervated by adrenergic nerves. Am J Physiol Heart Circ Physiol.

[48] Telinius N., Kim S., Pilegaard H. (2014). The contribution of K(+) channels to human thoracic duct contractility. Am J Physiol Heart Circ Physiol.

[49] Schumacher K.R., Cools M., Goldstein B.H. (2011). Oral budesonide treatment for protein-losing enteropathy in Fontan-palliated patients. Pediatr Cardiol.

[50] Kewcharoen J., Mekraksakit P., Limpruttidham N. (2020). Budesonide for protein losing enteropathy in patients with fontan circulation: a systematic review and meta-analysis. World J Pediatr Congenit Heart Surg.

[51] Schleiger A., Ovroutski S., Peters B. (2020). Treatment strategies for protein-losing enteropathy in Fontan-palliated patients. Cardiol Young.

[52] Roberts R.O., Di Maria M.V., Brigham D. (2020). Evidence of systemic absorption of enteral budesonide in patients with fontan-associated protein-losing enteropathy. Pediatr Cardiol.

[53] John A.S., Driscoll D.J., Warnes C.A. (2011). The use of oral budesonide in adolescents and adults with protein-losing enteropathy after the Fontan operation. Ann Thorac Surg.

[54] Uzun O., Wong J.K., Bhole V. (2006). Resolution of protein-losing enteropathy and normalization of mesenteric Doppler flow with sildenafil after Fontan. Ann Thorac Surg.

[55] Goldberg D.J., Zak V., Goldstein B.H. (2020). Results of the FUEL trial. Circulation.

[56] Agnoletti G., Gala S., Ferroni F. (2017). Endothelin inhibitors lower pulmonary vascular resistance and improve functional capacity in patients with Fontan circulation. J Thorac Cardiovasc Surg.

[57] Reinhardt Z., Uzun O., Bhole V. (2010). Sildenafil in the management of the failing Fontan circulation. Cardiol Young.

[58] Weingarten A.J., Menachem J.N., Smith C.A. (2019). Usefulness of midodrine in protein-losing enteropathy. J Heart Lung Transplant.

[59] Donnelly J.P., Rosenthal A., Castle V.P. (1997). Reversal of protein-losing enteropathy with heparin therapy in three patients with univentricular hearts and Fontan palliation. J Pediatr.

[60] Kelly A.M., Feldt R.H., Driscoll D.J. (1998). Use of heparin in the treatment of protein-losing enteropathy after fontan operation for complex congenital heart disease. Mayo Clin Proc.

[61] Ryerson L., Goldberg C., Rosenthal A. (2008). Usefulness of heparin therapy in protein-losing enteropathy associated with single ventricle palliation. Am J Cardiol.

[62] Bhagirath K.M., Tam J.W. (2007). Resolution of protein-losing enteropathy with low-molecular weight heparin in an adult patient with Fontan palliation. Ann Thorac Surg.

[63] Friedland-Little J.M., Gajarski R.J., Schumacher K.R. (2017). Dopamine as a potential rescue therapy for refractory protein-losing enteropathy in Fontan-palliated patients. Pediatr Transplant.

[64] John A.S., Phillips S.D., Driscoll D.J. (2011). The use of octreotide to successfully treat protein-losing enteropathy following the Fontan operation. Congenit Heart Dis.

[65] Vyas H., Driscoll D.J., Cabalka A.K. (2007). Results of transcatheter Fontan fenestration to treat protein losing enteropathy. Catheter Cardiovasc Interv.

[66] Rychik J., Rome J.J., Jacobs M.L. (1997). Late surgical fenestration for complications after the Fontan operation. Circulation.

[67] Hallbergson A., Mascio C.E., Rome J.J. (2015). Transcatheter fontan takedown. Catheter Cardiovasc Interv.

[68] Marathe S.P., Iyengar A.J., Betts K.S. (2021). Long-term outcomes following Fontan takedown in Australia and New Zealand. J Thorac Cardiovasc Surg.

[69] Hraska V., Hjortdal V.E., Dori Y. (2021). Innominate vein turn-down procedure: Killing two birds with one stone. JTCVS Tech.

[70] Brizard C.P., Lane G.K., Alex G. (2016). Original surgical procedure for the treatment of protein-losing enteropathy in fontan patients: report of two midterm successes. Circulation.

[71] Schumacher K.R., Yu S., Butts R. (2019). Fontan-associated protein-losing enteropathy and post‒heart transplant outcomes: a multicenter study. J Heart Lung Transplant.

[72] Amdani S., Simpson K.E., Thrush P. (2021 Feb 18). Hepatorenal dysfunction assessment with the Model for End-Stage Liver Disease Excluding INR score predicts worse survival after heart transplant in pediatric Fontan patients. J Thorac Cardiovasc Surg.

[73] Schumacher K.R., Gossett J., Guleserian K. (2015). Fontan-associated protein-losing enteropathy and heart transplant: a Pediatric Heart Transplant Study analysis. J Heart Lung Transplant.

[74] Simpson K.E., Pruitt E., Kirklin J.K. (2017). Fontan patient survival after pediatric heart transplantation has improved in the current era. Ann Thorac Surg.

[75] Mahle W.T., Tresler M.A., Edens R.E. (2011). Allosensitization and outcomes in pediatric heart transplantation. J Heart Lung Transplant.

[76] Magdo H.S., Stillwell T.L., Greenhawt M.J. (2015). Immune abnormalities in fontan protein-losing enteropathy: a case-control study. J Pediatr.

[77] Chavhan G.B., Amaral J.G., Temple M. (2017). MR lymphangiography in children: technique and potential applications. Radiographics.

[78] Ghosh R.M., Griffis H.M., Glatz A.C. (2020). Prevalence and cause of early fontan complications: does the lymphatic circulation play a role?. J Am Heart Assoc.

[79] Dori Y., Keller M.S., Fogel M.A. (2014). MRI of lymphatic abnormalities after functional single-ventricle palliation surgery. AJR Am J Roentgenol.

[80] Dori Y., Zviman M.M., Itkin M. (2014). Dynamic contrast-enhanced MR lymphangiography: feasibility study in swine. Radiology.

[81] Biko D.M., Smith C.L., Otero H.J. (2019). Intrahepatic dynamic contrast MR lymphangiography: initial experience with a new technique for the assessment of liver lymphatics. Eur Radiol.

[82] Smith C.L., Liu M., Saravanan M. (2022 Jan). Liver lymphatic anatomy and role in systemic lymphatic disease. Eur Radiol.

[83] Dori Y., Smith C.L., DeWitt A.G. (2020). Intramesenteric dynamic contrast pediatric MR lymphangiography: initial experience and comparison with intranodal and intrahepatic MR lymphangiography. Eur Radiol.

[84] Kylat R.I., Witte M.H., Barber B.J. (2019). Resolution of protein-losing enteropathy after congenital heart disease repair by selective lymphatic embolization. Pediatr Gastroenterol Hepatol Nutr.

[85] Al Balushi A., Mackie A.S. (2019). Protein-losing enteropathy following fontan palliation. Can J Cardiol.

[86] Smith C.L., Hoffman T.M., Dori Y. (2020). Decompression of the thoracic duct: a novel transcatheter approach. Catheter Cardiovasc Interv.

[87] Hraška V. (2013). Decompression of thoracic duct: new approach for the treatment of failing Fontan. Ann Thorac Surg.

[88] Kreutzer C., Klinger D.A., Chiostri B. (2020). Lymphatic decompression concomitant with fontan/Kreutzer procedure: early experience. World J Pediatr Congenit Heart Surg.

[89] Hraska V., Mitchell M.E., Woods R.K. (2020). Innominate vein turn-down procedure for failing fontan circulation. Semin Thorac Cardiovasc Surg Pediatr Card Surg Annu.

[90] Rychik J., Dodds K.M., Goldberg D. (2020). Protein losing enteropathy after fontan operation: glimpses of clarity through the lifting fog. World J Pediatr Congenit Heart Surg.

[91] Rychik J., Goldberg D., Rand E. (2013). End-organ consequences of the Fontan operation: liver fibrosis, protein-losing enteropathy and plastic bronchitis. Cardiol Young.

[92] Diller G.P., Arvanitaki A., Opotowsky A.R. (2021). Lifespan perspective on congenital heart disease research: JACC state-of-the-art review. J Am Coll Cardiol.

[93] Alsaied T., Allen K.Y., Anderson J.B. (2020). The Fontan outcomes network: first steps towards building a lifespan registry for individuals with Fontan circulation in the United States. Cardiol Young.

[94] Daley M., du Plessis K., Zannino D. (2020). Reintervention and survival in 1428 patients in the Australian and New Zealand fontan registry. Heart.

[95] Gatzoulis M.A. (2006). Adult congenital heart disease: education, education, education. Nat Clin Pract Cardiovasc Med.

[96] Fragata J.I.G. (2020). Lymphatic decompression for fontan/Kreutzer procedures-indeed, a clever concept, but will it truly work in practice?. World J Pediatr Congenit Heart Surg.

