# Supplementary figures and images for: Protein losing enteropathy after the Fontan operation
Source: Int J Cardiol Congenit Heart Dis. 2022 Jan 26;7:100338. doi: 10.1016/j.ijcchd.2022.100338 (PMC11657892; doi:10.1016/j.ijcchd.2022.100338)

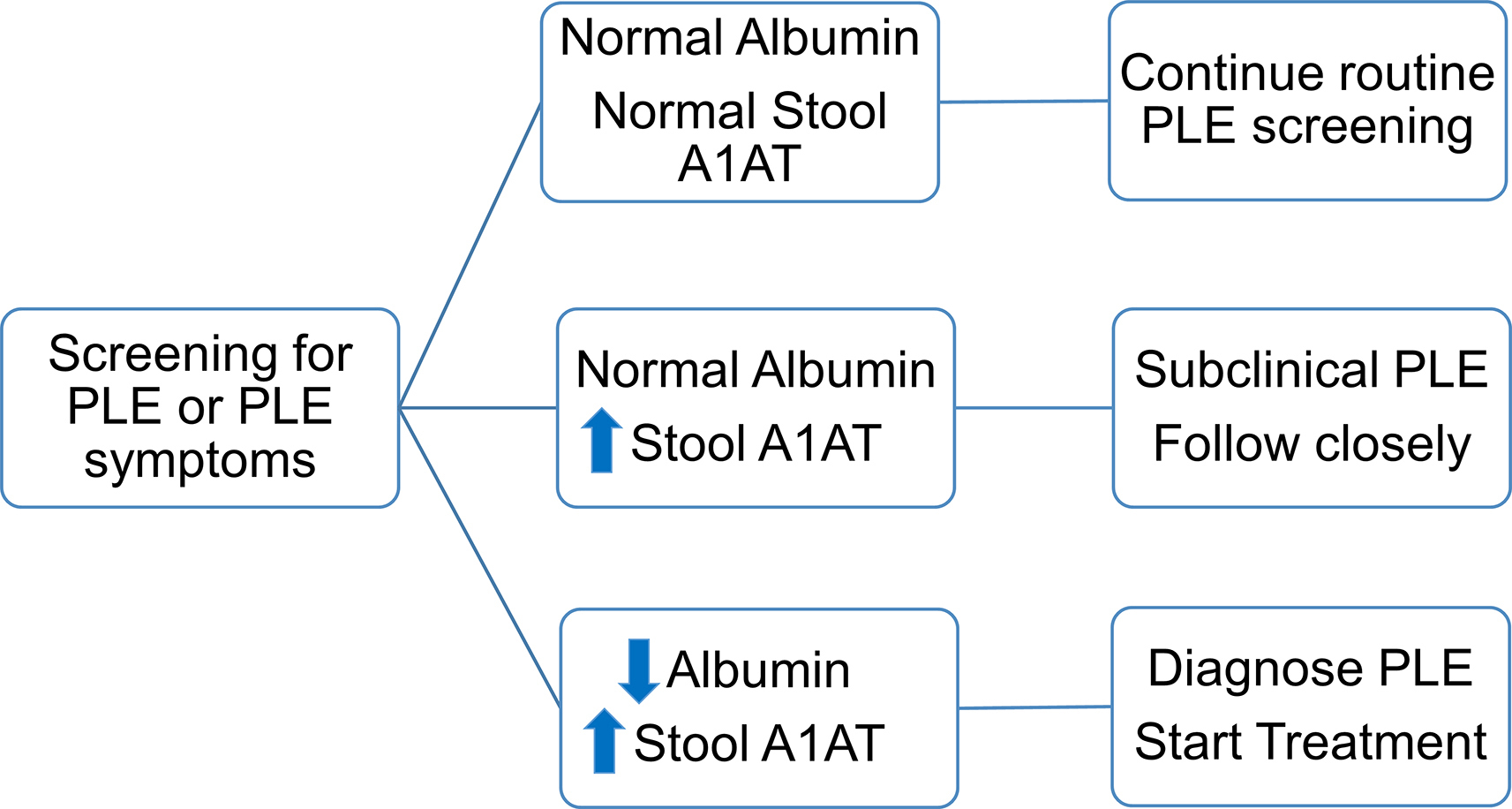

Supplement: figs1 [file mmcfigs1.jpg]

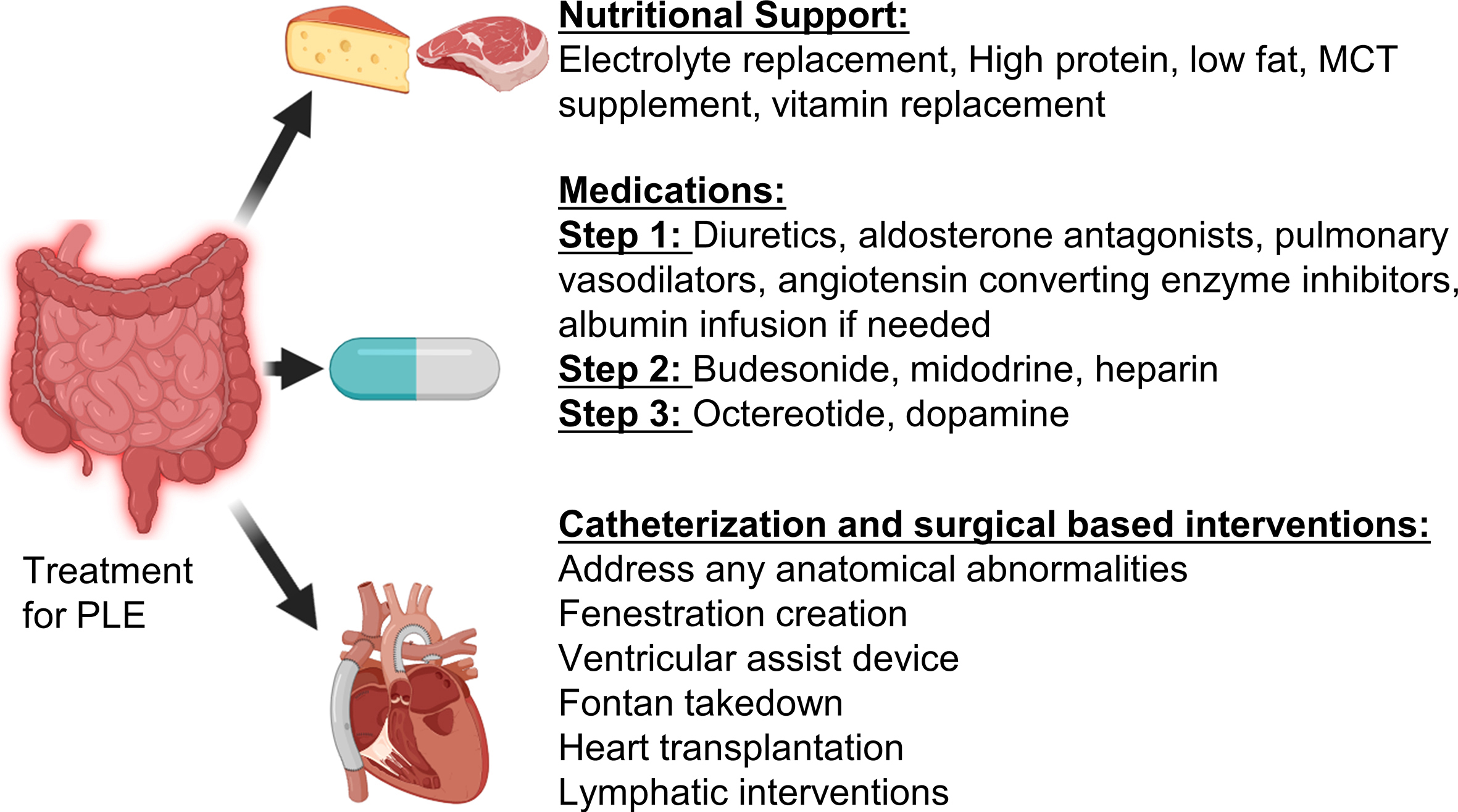

Supplement: figs2 [file mmcfigs2.jpg]
